# Evaluation of Novel Concentrated Interdisciplinary Group Rehabilitation for Patients With Chronic Illnesses: Protocol for a Nonrandomized Clinical Intervention Study

**DOI:** 10.2196/32216

**Published:** 2021-10-07

**Authors:** Gerd Kvale, Bente Frisk, Marte Jürgensen, Tore Børtveit, Øystein Theodor Ødegaard-Olsen, Ane Wilhelmsen-Langeland, Bernt Bøgvald Aarli, Kristina Sandnes, Sidsel Rykken, Anne Haugstvedt, Sigurd William Hystad, Eirik Søfteland

**Affiliations:** 1 Helse i Hardanger Øystese Norway; 2 Department of Clinical Psychology University of Bergen Bergen Norway; 3 Division of Psychiatry Haukeland University Hospital Bergen Norway; 4 Faculty of Health and Social Sciences Western Norway University of Applied Sciences Bergen Norway; 5 Department of Physical Medicine and Rehabilitation Haukeland University Hospital Bergen Norway; 6 Department of Thoracic Medicine Haukeland University Hospital Bergen Norway; 7 Department of Clinical Science University of Bergen Bergen Norway; 8 Department of Psychosocial Science University of Bergen Bergen Norway; 9 Department of Medicine Haukeland University Hospital Bergen Norway

**Keywords:** COVID-19, chronic illnesses, concentrated rehabilitation, low back pain, post–COVID-19 symptoms, post–COVID-19 syndrome, long COVID, fatigue, type 2 diabetes, anxiety, depression

## Abstract

**Background:**

An aging population with a growing burden of chronic complex illnesses will seriously challenge the public health care system. Consequently, novel and efficacious treatment approaches are highly warranted. Based on our experiences with concentrated treatment formats for other health challenges, we developed a highly concentrated interdisciplinary group rehabilitation approach for chronic illnesses.

**Objective:**

We aim to explore the acceptability of the intervention and describe potential changes in functional impairment at follow-up.

**Methods:**

The cornerstones of the intervention are as follows: (1) prepare the patient for change prior to treatment, (2) focus on health promoting microchoices instead of symptoms, and (3) expect the patient to integrate the changes in everyday living with limited hands-on follow-up. The intervention will be delivered to patients with highly diverse primary symptoms, namely patients with low back pain, post–COVID-19 symptoms, anxiety and depression, and type 2 diabetes.

**Results:**

Recruitment started between August 2020 and January 2021 (according to the illness category). For initial 3-month results, recruitment is expected to be completed by the end of 2021.

**Conclusions:**

If successful, this study may have a substantial impact on the treatment of low back pain, post–COVID-19 symptoms, anxiety and depression, and type 2 diabetes, which together constitute a major socioeconomic cost. Further, the study may widen the evidence base for the use of the concentrated treatment format in a diverse group of medical conditions.

**International Registered Report Identifier (IRRID):**

DERR1-10.2196/32216

## Introduction

### Background

An aging population with a growing burden of chronic complex diseases seriously challenges the ability to deliver adequate health services [1-3]. Adding to this, the ongoing COVID-19 pandemic has resulted in a new group of patients, who, having recovered from the acute infection, may experience a range of long-lasting symptoms. This hitherto unknown condition is frequently termed post–COVID-19 syndrome (or long COVID) and may also affect people in whom the primary infection was mild [4,5]. If we do not succeed in delivering treatment that enables patients to deal effectively with their long-lasting illnesses, the public health care system is unlikely to cope with the upcoming demographic challenges [6,7].

Although patients with chronic illnesses have a wide range of symptoms, pain and fatigue are among the most common [8,9]. Medical advice typically encourages the patient to be as active as possible, eat healthily, get enough sleep, avoid stress [10-12], and monitor improvement or worsening by using medical diaries and symptom logs [13]. Since the patient’s main concern is to prevent worsening of the condition, activities that might increase symptoms are typically avoided [14]. Examples of such behavior patterns can be to restrict physical activity upon muscle pain; to stand, walk, or move carefully; to rest or sleep whenever feeling exhausted or tired; and to rest before or after engaging in activities. Over time, such coping strategies are likely detrimental and might contribute to conservation or worsening of symptoms. This is especially the case when the first indication of improvement might be a temporary worsening of symptoms, such as muscle pain or tiredness after increased physical activity.

Based on extensive experience with concentrated treatment formats [15-19], we have developed a comprehensive transdiagnostic rehabilitation for chronic illnesses, characterized by a systematic focus on how to initiate and maintain change. In the presently described protocol, this intervention will be piloted on patients with a diversity of chronic health challenges, including chronic low back pain, post–COVID-19 symptoms, anxiety and depression, and type 2 diabetes. These conditions were chosen as they collectively represent major personal and societal costs [20]. Furthermore, the included disorders are characterized by fundamentally different symptoms and challenges (eg, pain, fatigue, depression, anxiety, dyspnea, and glucose variability). In consequence, we are able to summarize the overall effectiveness of the intervention across disorders, in addition to illness-specific outcomes. Finally, by including patients with post–COVID-19 symptoms (fatigue, dyspnea, problems with concentration, diurnal patterns, and/or nutrition), we may be able to further advance this field, as there presently are large knowledge gaps concerning the long-term prognosis and natural development of the complaints [21].

One of the main features of this novel cross-disciplinary concentrated intervention (lasting less than a week) is a shift in focus from targeting symptoms to targeting and monitoring everyday microchoices that facilitate increased levels of functioning. The intention of these microchoices is to break inflexible patterns of symptom regulation by “doing something different” whenever tempted to be guided by the symptoms. This approach enables the patient to systematically increase flexibility and their levels of functioning when symptoms and health challenges are present. In addition, a focus on deliberate behavior instead of symptoms implies that change is within reach and possible to control [17,22].

In order to ensure a safe setting in which participants may challenge their current coping strategies, they will work together with an interdisciplinary team, and each patient will design individually tailored plans for the most relevant microchoices. To ensure that the patients are prepared to initiate change, they are thoroughly introduced to the program prior to treatment, and if reluctant or unable to dedicate their full attention, they are encouraged to postpone participation until ready. After the concentrated intervention, the patients will be prepared to integrate the changes as part of their everyday living.

The aim of this pilot study is to explore the acceptability of the concentrated interdisciplinary group rehabilitation for patients with chronic low back pain, post–COVID-19 symptoms, anxiety and depression, and type 2 diabetes, and to describe the basic changes in functional status. Based on our experiences with other concentrated treatment formats, we expect the intervention to be highly acceptable and to have significant effects on functional impairment [15-19].

### Main Hypotheses

We hypothesize that the treatment will be acceptable as indicated by the following [22,23]:

1. Proportion (≥90%) of patients who meet the inclusion criteria and accept participation.
2. Proportion (≥90%) of included patients who attend the concentrated intervention.
3. Proportion (≥90%) of included patients who complete participation in the concentrated intervention.

Further, we hypothesize that patients will be satisfied with the treatment, as defined by a mean Client Satisfaction Questionnaire (CSQ) score of 20 or more, and with no single dimension below an average score ≤2. A cutoff score of 20 has been chosen based on previous research using scores ≥20 to indicate “good” satisfaction [24].

Finally, we hypothesize that the patients’ levels of functioning will be improved at follow-up as measured with the Work and Social Adjustment Scale (WSAS), and that there will be a significant change in how much the illness affects the patients’ life as measured with the Brief Illness Perception Questionnaire (BIPQ) in the following domains:

1. How much does your illness affect your life?
2. How much control do you feel you have over your illness?
3. How concerned are you about your illness?
4. How well do you feel you understand your illness?

We do not anticipate significant changes in the following domains for all 4 included illnesses:

1. How long do you think your illness will continue?
2. How much do you experience symptoms from your illness? How much do you think your treatment can help your illness?
3. How much does your illness affect you emotionally? (eg, does it make you angry, scared, upset, or depressed?)

## Methods

### Overview

This pilot study is part of the “Project Development of Smart Health Solutions” (PUSH project), a collaboration between Haukeland University Hospital (Bergen, Norway) and Helse i Hardanger (Øystese, Norway). The overall aim of the PUSH project is to develop more efficient and cost-effective treatments to be integrated as part of public health care. The project is headed by a steering committee at Haukeland University Hospital, whereas the interventions and study data collection are primarily done at Helse i Hardanger, a health care research facility located outside Bergen.

### Study Design and Participants

**Figure 1:**
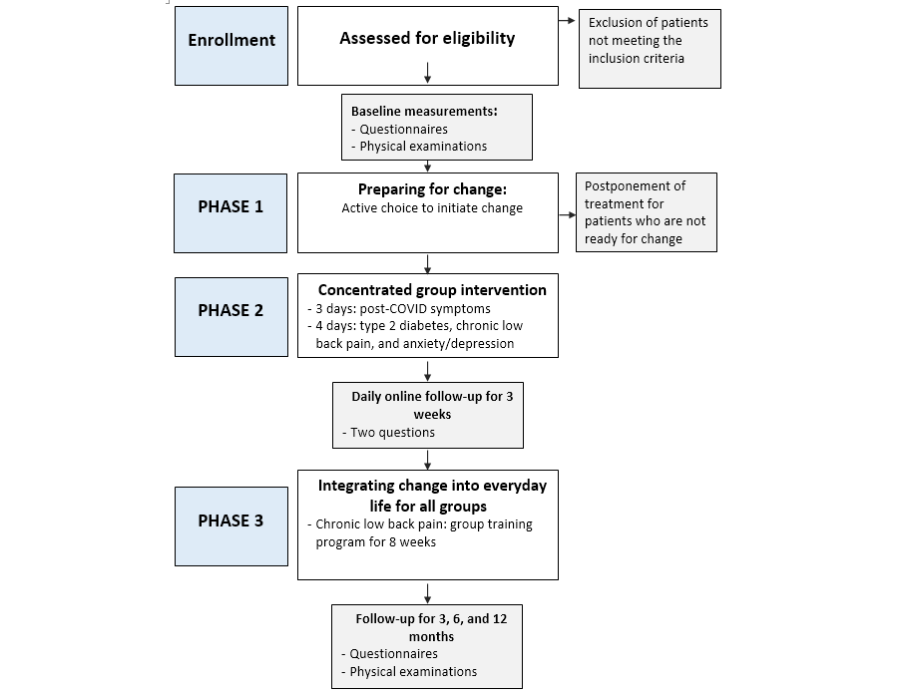
Flowchart of the study. With regard to illness-specific physical exercise tests and examinations, evaluation was performed with questionnaires at pretreatment, and 1-week and 3-, 6-, and 12-month follow-ups. The 2 questions were as follows: To what extent did you allow the symptoms to decide today (0-10) and To what extent did you make use of the principle of doing something else (0-10).

This study is designed to test the acceptability of concentrated interdisciplinary group rehabilitation with an open pre-post follow-up design in the following 4 groups of patients with chronic illnesses: chronic low back pain, post–COVID-19 symptoms, anxiety and depression, and type 2 diabetes. The treatment will be delivered in groups of 6 to 10 patients, and the initial pilot study will include between 40 and 50 patients for each illness (4-6 treatment groups for each illness). For the inclusion and exclusion criteria, please refer to Textbox 1, and for the overall study flowchart, please refer to Figure 1.

**Inclusion and exclusion criteria.**

**For all diagnoses**

***Inclusion criteria***

- Fluent in oral and written Norwegian
- Access to a smartphone and sufficient digital competence to handle online questionnaires
- Negative COVID-19 polymerase chain reaction test

***Exclusion criteria***

- Cognitive failure
- Lack of self-reliance in daily routine
- Severe mental health problems preventing engagement in the rehabilitation program
- Conditions that inhibit physical activity

**Chronic low back pain**

***Inclusion criteria***

- Low back pain with or without radiculopathy
- Age between 18 and 70 years
- Low back pain >3 months, and at least 4 months of sick leave
- Ability to participate in a group-based posttreatment physical training in Bergen, Voss, or Kvam municipalities

***Exclusion criteria***

- Surgery during the last 8 weeks
- Available alternative rehabilitation therapy for low back pain

**Type 2 diabetes**

***Inclusion criteria***

- Type 2 diabetes
- Age >18 years
- Presence of at least one of the following complications/challenges:
  - Dysglycemia
  - Frequent or severe hypoglycemia
  - Weight gain
  - Diabetes complications
  - Diet, physical activity, and/or medical treatment challenges

***Exclusion criteria***

- Type 1 diabetes
- Monogenic diabetes
- Secondary diabetes (pancreatogenic or any other form of secondary diabetes)
- Ongoing pregnancy

**Post–COVID-19 symptoms**

***Inclusion criteria***

- Age between 18 and 67 years
- >2 months since the COVID-19 infection
- Impaired ability to work full time
- Substantial post–COVID-19 physical and/or mental health problems
  - Fatigue
  - Dyspnea
  - Concentration difficulties
  - Sleeplessness, diurnal disturbances
  - Nutritional deficiencies

***Exclusion criteria***

- Exercise contraindication

**Anxiety or depression**

***Inclusion criteria***

- Age between 18 and 47 years
- Fulfilling ICD-11 criteria for one of the following disorders:
  - Mixed anxiety and depressive illness
  - Other mixed anxiety illnesses
  - Unspecified anxiety illness
  - Generalized anxiety illness
  - Depressive episode
  - Recurrent depression
  - Unspecified recurrent depression

***Exclusion criteria***

- Bipolar illness
- Psychosis
- Ongoing substance abuse/dependence
- Ongoing suicidal ideation

### Procedures and Patient Flow

Although patients themselves may initiate the process, all potential participants need to be referred by their general practitioner or other physician responsible for the treatment of the relevant condition. If the patient fulfills the inclusion criteria, they will be invited to sign the informed consent and offered participation in the project successively upon availability in the groups.

One of the clinicians will call the patients upon referral and check the inclusion/exclusion criteria. During this phone call, the patients will be informed about the PUSH project and that the intervention is a concentrated interdisciplinary group treatment that will take place in Øystese, outside Bergen. If they fulfill the inclusion criteria and none of the exclusion criteria, an appointment for screening will be made. Before they answer the online questionnaires, they will be asked to watch videos describing the program, to ensure that all participants receive the same information (at the homepage [25]). For low back pain and type 2 diabetes, the informed consent will be signed online, while patients with post–COVID-19 symptoms or anxiety/depression will sign at baseline testing, when the first face-to-face meeting takes place. For low back pain and type 2 diabetes, participants will be invited in groups for an approximately 2-hour meeting 1 to 3 weeks before the treatment to make sure they are prepared for the intervention. For patients with anxiety and depression, or post–COVID-19 symptoms, the same information will be provided individually during the screening. All patients will be contacted by a therapist during the week prior to the treatment to confirm that they have received all necessary information and are ready to start their concentrated rehabilitation.

### Outcomes

Assessments will be performed before and 1 week after the concentrated rehabilitation program, and after 3, 6, and 12 months. An overview of the measurement tools and the respective assessment times are presented in Table 1. The outcomes are selected with the aim of describing the overall experiences with the concept of the concentrated treatment format. More detailed disease-specific outcome measures will also be assessed, but will not be presented in this generic protocol paper, as they pertain to other aspects than the concentrated treatment format per se. The initial results will be published following 3 months of follow-up, whereas the final results are to be published upon 12 months of follow-up.

**Table 1:** Questionnaires and clinical and physical examinations.

Questionnaires and assessments	Assessment point
Client Satisfaction Questionnaire	One week after rehabilitation
Brief Illness Perception Questionnaire	Baseline, and 1 week and 3, 6, and 12 months after rehabilitation
Work and Social Adjustment Scale	Baseline, and 3, 6, and 12 months after rehabilitation
Strategies for handling symptoms (rating 0-100 and 2 questions^a^)	Daily reports for 21 days after rehabilitation
Bergen Insomnia Scale	Baseline, and 1 week and 3, 6, and 12 months after rehabilitation
Patient Activation Measure	Baseline, and 1 week and 3, 6, and 12 months after rehabilitation
EQ-5D-5L (EuroQOL)	Baseline, and 3, 6, and 12 months after rehabilitation
Consumption of health care	Baseline, and 3, 6, and 12 months after rehabilitation
Medication lists	Baseline, and 3, 6, and 12 months after rehabilitation
Body weight/BMI	Baseline, and 3, 6, and 12 months after rehabilitation

^a^To what extent did you allow the symptoms to decide today (0-10) and To what extent did you make use of the principle of doing something else (0-10).

#### Primary Outcome Measures

##### Acceptability

The acceptability of the treatment will be measured by the following variables: (1) The proportion of patients accepting to participate in the treatment among those fulfilling the inclusion criteria and offered participation; (2) The proportion of patients offered participation who start treatment; and (3) The proportion of patients completing the treatment program (on-site).

##### CSQ

The CSQ-8 is an 8-item questionnaire that measures patient satisfaction with health services, where the items are rated from 1 (very low satisfaction) to 4 (very high satisfaction) [26]. The total score ranges from 8 to 32, with higher scores indicating higher degrees of satisfaction. The CSQ-8 has good psychometric properties, with high internal consistency (Cronbach α=.93) and high interitem correlation [27].

##### BIPQ

The BIPQ is a 9-item questionnaire designed to assess cognitive and emotional representations of illness [28]. Questions are graded from 1 to 10. The last item deals with the perceived cause of illness, in which respondents list the perceived 3 most important causal factors in their illness. For this questionnaire, the general word “illness” can be replaced by the name of a particular illness. The word “treatment” in the treatment control item can be replaced by a particular treatment such as “surgery” or “physiotherapy.” The scale has good psychometric properties according to a recent review [29].

##### WSAS

The WSAS is a short questionnaire measuring the impact of the illness on aspects of work and social activities [30]. The scale consists of five items rated from 0 (not at all) to 8 (very severe), and a higher score indicates higher impairment (maximum score is 40). The scale is regarded as reliable and valid, with good psychometric properties.

#### Transdiagnostic Secondary Outcome Measures

At pretreatment and posttreatment, and the follow-up assessments, the patients will be asked to rate on a scale from 0 to 100 to what extent they use the following strategies when trying to handle the symptoms: (1) Wait to start an activity until I feel up to it; (2) Wait to start an activity until I am certain that I will succeed; (3) Ensure that the symptoms will not get worse; (4) Ensure that I am prepared to handle challenges; (5) Try to calm down before proceeding when I get anxious; (6) Spend a lot of time on worrying and ruminating; (7) Avoid socializing if I do not feel up to it; (8) Ensure that I get enough rest; (9) Try to not let others see how I feel; (10) Try to have a positive mindset; and (11) Follow my gut feeling. The questionnaire was developed in cooperation with patients having previous experience in the concentrated treatment format.

### Intervention

The intervention consists of the following 3 equally important phases (Figure 1): (1) Preparing for change, (2) The concentrated intervention, and (3) Integrating change into everyday living. Throughout the intervention, the focus is on how to initiate and maintain change by utilizing discomfort as a guide to break inflexible patterns of symptom regulation. By intention, the topics introduced in the different phases overlap considerably. In order to incorporate the central aspects of a given health challenge, minor illness-specific adaptations are made to the intervention.

#### Phase 1: Preparing for Change

It is essential that the patients are thoroughly informed and prepared prior to the rehabilitation, and that they have made an active choice to initiate change. During the pretreatment information meeting, the topics below will be covered using nontechnical terms and easily understandable metaphors (with slight illness-relevant modifications).

##### Improved Everyday Functioning

The goal of this program is to help the participant to live a life where the symptoms/health challenges do not decide how the person behaves. Thus, it is a program focused on change, with the goal of a better life and improved everyday functioning.

##### Challenge Patterns of Symptom Regulation

Living with a chronic illness implies that patients are continuously trying to prevent their health from getting worse, which makes sense. However, one of the consequences might be that the patients develop patterns of symptom regulation that might contribute to conservation or, in some instances, might exacerbate the problem. Typically, the patient finds it hard to know when to be cautious and when to challenge a given strategy to deal with the symptoms. In our experience, people with chronic illnesses typically have an adequate understanding of their problems, but this does not necessarily lead to change. It may rather increase the feeling of helplessness because they do not know how to initiate and sustain change. The concentrated treatment is a practical deliberate approach focused on how to identify and break unhelpful patterns of symptom regulation.

##### Therapist-Assisted Behavior Activation

In line with the above, the illness is frequently a composite of health challenges that might require expertise from a number of specialized professions. During 4 consecutive days (3 for post–COVID-19 symptoms), a highly qualified interdisciplinary team will provide practical information and hands-on coaching while the patients challenge the way symptoms are handled. Patients referred to the program for anxiety and depression are, prior to treatment, expected to provide suggestions regarding unhelpful patterns of regulation, which they are willing to start changing during the concentrated treatment days (eg, avoidance of specific situations, social withdrawal, etc).

##### Group Setting

The concentrated treatment is delivered in groups of 6 to 10 patients. The participants will work side by side and challenge their own expectations regarding what they are capable of doing. In the group setting, each participant will need to take responsibility for making the treatment sessions relevant for their specific problems.

##### Basic Bodily Rhythms

An important aspect of the treatment is a focus on the practical implications of bodily rhythms such as sleep-wakefulness, activity-rest, and meal habits.

##### Substantial Self-Effort

All participants are expected to stay at the adjacent hotel throughout the treatment week in order to dedicate full days to the treatment and engage in all parts of the program from 8:30 AM to 4:00 PM. On the last day, the program will be finished after lunch. From 4:00 PM to 7:00 PM, each participant will practice on their own based on what they learned during the treatment. This means that there will not be room for other appointments during the concentrated intervention period, and their full focus will be on changing their unhelpful behavior patterns.

##### Start Planning Life After the Concentrated Intervention 

Since the focus is to increase the level of functioning, it is necessary that each participant, prior to the treatment week, decides upon how to practice the changes and integrate them into their lives, starting directly after returning home. This includes specific plans to increase participation at work/school or other activities that will lead to better daily functioning.

##### No Participation Until Ready

No treatment or medicine works if the patient is not willing to take it. This is even true for penicillin. If the patients are reluctant to participate, we will recommend waiting until they are ready to make a change.

An adapted version of the Borkovec and Nau *Reaction to Treatment Scale* is used to explore the patients’ readiness for treatment [31]. A low rating (<70%) on any of the 4 questions (“How much does this approach make sense?” “How likely is it that you would recommended this treatment to a friend with similar problems?” How likely is it that you will be fully engaged in the program?” “How likely do you think it is that you will benefit from the program?”) will serve as an opportunity to clear up any misunderstandings and, together with the patient, decide if it might be better to postpone treatment initiation.

#### Phase 2: The Concentrated Group Intervention

The program starts with patient education, and the most important points will be repeated throughout the week. At the first session, rules of confidentiality will be established. During the first part of day 1, each participant will provide some information about his/her health problem based on the following: “How long have you struggled with this health challenge?” “What does it prevent you from doing?” “What are you looking forward to do when this is no longer a problem?” The participants will use 2 to 3 minutes each. Patient education will be interspaced with physical activity, brief mindfulness sessions, and practical training sessions focused on breaking problematic patterns of symptom regulation

##### Transdiagnostic Elements of the Group Intervention

###### Patient Education

Patient education will be provided on how to initiate and maintain relevant change and, at the same time, accept those things that cannot be changed or controlled (eg, history, thoughts, and feelings [for post–COVID-19 symptoms and type 2 diabetes: getting the infection/having the illness]). It will be underscored that change starts with an active decision and that the goal of the treatment is to increase flexibility and to live a life where the symptoms do not decide.

###### Microchoices

Microchoices will be used as a term that refers to the moments when you discover specifically how and where in your everyday life the symptoms are making choices on behalf of you, and where you have an option to choose differently. Participants will be encouraged to do things they have avoided in fear of symptom worsening. It will be emphasized that change is measured in behavior (what you do) and *not* in the reduction of symptoms. Symptom reduction, on the other hand, will be described as a positive and valuable side effect of behavioral change. This shift in focus from symptoms to deliberate behavior implies that change is within reach. Furthermore, participants will be challenged to do a value-based microchoice each day, for example, call a friend or relative whom they had neglected due to the health problems. During patient education, this concept is introduced and explained (ie, having health problems and symptoms may make people more self-centered and lose perspective, making them lose track of who they were, and value-based actions may help them get back on track and widen their focus).

###### Individually Tailored Practice

Individually tailored practice in discovering microchoices and “breaking problematic patterns” in as many relevant settings as possible will be the cornerstone of each day.

###### Feedback and Coaching

Each morning, at lunchtime, and at the end of the joint program, everyone will be asked to share their self-evaluation on how successful they have been in identifying their own patterns of symptom regulation, and to what extent they have attempted to break the patterns. A scale from 1 to 6 will be used. If they rate themselves lower than a 6, the group leader will explore the reasons for this, with the aim of helping them find the moments of “microchoices” and identify what they can do to break the pattern. Throughout these sessions, symptoms will not be given attention, but rather described as being important, in order to be able to identify targets where it might be possible to break the patterns of regulation.

###### Physical Activity

These sessions will vary across illnesses; however, common for all participants will be instructions to attempt making the physical activity relevant for their own challenges and fit into their projects of “breaking patterns of symptom regulation.” For some, this might mean to refrain from the temptation to overdo, and for others, it might imply to be more active or active in a different way. For patients with anxiety/depression, the task during the physical activity will be to “surprise themselves” by doing a little more when they feel they have reached their limit. Patients with low back pain will follow the validated ready-to-use program “GLA:D Back,” integrating patient education and exercise therapy [32,33]. For post–COVID-19 patients, the physical training will be a mix of high- and low-intensity training, focusing on increased exercise capacity and the restoration of trust in one’s own body. In the type 2 diabetes group, the main aim will be to experience how a diverse range of activities can be useful in order to maximize the effect of the body’s available insulin.

###### Mindfulness

Each day will contain one to three brief sessions of detached mindfulness, where the task will be to focus on breathing, while at the same time observe (and accept, without trying to change) wandering thoughts, bodily sensations, etc [34].

###### Food and Meal Habits

One of the patient education sessions will be focused on useful dietary choices. For patients with post–COVID-19 symptoms affecting diet and/or nutrition, the focus will be on useful dietary choices with a focus on helping the body to recover. In patients with anxiety and depression, the focus will be on the establishment of good mealtime habits as a way of restoring diurnal rhythms. For patients with type 2 diabetes, the emphasis will be to explore how to get the maximum out of the available insulin (ie, their beta-cell capacity or their insulin injections). During this session, they will also test different “forbidden” (carbohydrate-rich) food items and evaluate the consequences on their blood glucose levels. This will be followed by physical activity to experience the restoration of habitual glucose levels. The aim is to recognize that no food is forbidden, but that there are consequences of the choices, with various possible compensatory actions.

###### Pharmacist

For all participants, a pharmacist will review the individual medication lists, focusing on potential harmful interactions. In the low back pain group, 2 sessions of patient education are provided. The first session is on various types of formulations and medications in general, followed by an illness-specific session focusing on the risks of combining pain medications, the potential impact on driving capabilities, and an overall emphasis on minimizing the use of opioid-based and other pain-relief symptom treatments. Individual advice on the downsizing and discontinuation of medication is provided in cooperation with the study physician. Following the same pattern, the type 2 diabetes group also has 2 interactive educative sessions with the pharmacist. Here, the second session deals specifically with diabetes medications (use, effects, and most common side effects). For patients with post–COVID-19 symptoms, a short patient education is followed by a brief counseling session with a pharmacist on the proper use of inhalators.

###### Afternoon Practice

Before the group splits at 4:00 PM each day, individual practice plans for each afternoon and evening will be made, focusing on implementing microchoices in terms of physical activity, social/value-based activity, self-reflection, etc.

###### Individual Consultation

During the program, all patients will receive an individual consultation with the group leader or one of the psychologists or psychiatrists who are experts on the concentrated treatment format. This consultation will be focused on how to integrate the change into everyday living.

###### Preparing for Life After the Treatment

By the end of the program, all patients will have made a specified plan addressing how to integrate the change into normal living using the concept of SMART (specific, measurable, achievable, relevant, time-bound) goals [35,36]. Moreover, they will start answering the following daily questions online: “To what extent did you allow the symptoms to decide today” (0-10) and “To what extent did you make use of the principle of *doing something else*” (0-10).

#### Phase 3: Integrating the Change Into Everyday Living

##### Daily Online Reports

For 3 weeks after the concentrated program, through a digital solution, all patients will answer the 2 daily questions (described above) pertaining to the maintenance of the change. This will be done without feedback from the program. Helse i Hardanger is in the process of developing a smartphone app through which the patients are expected to answer questionnaires, store their SMART goals, and provide feedback. The program will facilitate contact with the clinic if needed.

##### Individual Video or Phone Consultation

Ten days after the concentrated treatment, an individual phone or video consultation is performed, focusing on how to maintain the change. Additionally, patients with low back pain follow the program for GLA:D Back, with training twice a week for 8 weeks at certified local GLA:D Back facilities.

##### Further Follow-Up and Data Gathering

All patients will answer questionnaires at 3-, 6-, and 12-month follow-ups. Further disease-specific examinations and/or data may also be gathered; however, the descriptions of these are outside the scope of this generic protocol paper.

### Competency in the Concentrated Treatment Format

A psychologist with extensive experience with concentrated treatment formats will lead all groups for patients with anxiety/depression, and these groups will be used for hands-on training in the format for clinicians working with the other illnesses. The content of all manuals (standard operating procedures) is supervised and approved by the originator of the format (GK), and all groups will receive hands-on supervision daily from GK.

### Statistical Analyses and Data Handling

Data will be analyzed with Stata version 17 (StataCorp). Changes in WSAS and BIPQ scores from pretreatment to posttreatment and the 3-month follow-up will be examined with repeated measures analyses. Statistical significance will be set at α=.05. Within-group effect sizes will be calculated using Glass’ Δ, with pretreatment SD as the denominator. Glass’ Δ is the recommended effect size for intervention studies, in which there are reasons to believe that the treatment will influence the SD as well as the mean [37]. An effect size is commonly interpreted as small (0.2), moderate (0.5), or large (0.8). Considering that this project is a pilot study of a novel interdisciplinary group treatment and that both the WSAS and BIPQ are global and not condition-specific measures, we expect the effect sizes to be small to moderate in magnitude.

Missing data on the primary outcome variables will be handled by multiple imputation (MI). Under the *missing at random* (MAR) assumption, MI is currently one of the best available methods of dealing with missing data and will provide unbiased estimates [38]. The main analyses of the primary outcomes will thus be conducted under the assumption of MAR. Sensitivity analyses will be conducted to assess the robustness of the results and the potential impact that nonignorable missingness may have on the estimated results. These sensitivity analyses will take a pattern-mixture approach [39]. In short, a pattern-mixture approach can involve assuming that participants who are lost to follow-up have a mean outcome that differs from that of participants who do not drop out by an offset. The impact on the results of various choices of clinically plausible offsets can then be examined, and if the effect from the primary analysis is qualitatively maintained for the range of plausible offsets, the findings can be said to be robust.

#### Data Collection and Monitoring

For each illness, a centralized team will be established. This team will have monthly contact with a designated researcher for each illness in order to monitor inclusion and data collection. Most of the data will be collected electronically, and all sensitive data will be stored on an encrypted server at Helse Vest IKT. Once the patients are included, all data entered by them will be monitored by the Study Administrative Team, as part of clinical follow-up.

### Adverse Events

If an acute condition occurs, the patients will receive the necessary care, and they might be excluded from the study if there are concerns about safety. Such patients will be thoroughly described and accounted for, in line with illness-specific standard operating procedures.

### User Involvement

The project has established a broad user panel with representatives recruited through Haukeland University Hospital and Helse i Hardanger. The following organizations are represented: Norwegian Asthma and Allergy Association, Norwegian Rheumatics’ Association, Mental Health Norway, Breast Cancer Association, Norwegian Diabetes Association, Norwegian Association for Lung and Heart Disease, and “Grannehjelpa” (neighbors’ help). The panel has given feedback throughout the development of the protocol and has approved the final version.

### Ethical Considerations

The PUSH project and the web application have been approved by the Research Ethical Board, Helse Vest (2020/101638), and the project will be conducted in accordance with the Helsinki Principles.

### Gender Perspectives

The inclusion factors and all interventions are gender neutral. However, in order to ensure adequate external validity and proper representation, we have no absolute limits in terms of the minimum inclusion rates of one gender. For all illnesses, the project will aim for at least 30 to 70 representations of the genders.

## Results

Recruitment started between August 2020 (anxiety/depression) and January 2021 (diabetes). For initial 3-month results, recruitment is expected to be completed by the end of 2021.

## Discussion

### Overview

In this paper, we describe a protocol for the establishment and initial evaluation of a novel concentrated interdisciplinary group rehabilitation for patients with chronic low back pain, post–COVID-19 symptoms, anxiety/depression, or type 2 diabetes. To our knowledge, this will be the first study to evaluate concentrated interdisciplinary group rehabilitation for such a diverse range of chronic health issues. Based on previous experiences with the short concentrated treatment format, we hypothesize that the intervention described will be positively received by the participants, will reduce the impact of the illness on their lives, and will improve the level of daily functioning.

The study is designed with participants as their own controls (pre-post comparisons) with a 12-month follow-up. Although this allows for summarizing the experiences and findings as described above, causal conclusions on the effects of the intervention may not be drawn. However, in our mind, this is an essential first step enabling subsequent separate controlled trials where such research questions, as well as cost-effectiveness issues, may be addressed. Still, the modest labor factor (10 patients, 2 group leaders) clearly benefits our concentrated treatment format, if shown to be efficacious.

If the intervention is followed by meaningful improvements for the participants, we will be able to help large groups of patients with chronic illnesses to adhere to choices in their daily living that eventually will enhance their functional status. The fact that participants need only 3 or 4 days of sick leave to take part in the intervention owing to the concentrated format, with the continued rehabilitation process at home, is another clear advantage. Existing rehabilitation programs for chronic illnesses often have a duration of 3 to 4 weeks or longer and therefore require a longer time away from home and, for those who are working, longer sick leave. Another benefit of our intervention is the focus on implementing microchoices in everyday life, with guided practice during the intervention and assisted introduction into life at home and work. Although this needs to be investigated, the aim is to ensure the long-term maintenance of the new life trajectory.

Considering the methodology, dropouts and poor adherence to the intervention can threaten the internal validity. Although proper information aims to limit this problem, we cannot be sure that the participants will participate for the whole period or whether they will complete digital and clinical examinations at 3-, 6-, and 12-month follow-ups. A notice will be sent to the patients about 2 to 3 weeks before the assessments. Participants who do not answer the questionnaires online or do not show up for clinical follow-up will be contacted by telephone. The impact of missing data will be assessed with appropriate statistical methods (ie, multiple imputations and sensitivity analysis). Finally, although the acceptability of the treatment, as defined in this project, has been used in a number of publications with a concentrated treatment format, this outcome is not formally validated [22,23]. To compensate, all causes for not accepting, attending, or completing the intervention will be recorded.

This protocol describes a novel transdiagnostic rehabilitation approach, and the illnesses in focus are clearly disparate. Hence, if the intervention in the described study appears effective, it could trigger new studies investigating the effects on other chronic diseases or health challenges. In line with this and to facilitate potential dissemination of the concentrated rehabilitation format, a training program for relevant health professionals will be made available.

### Conclusion

We present a protocol for the establishment and evaluation of a novel concentrated treatment to be investigated in patients with chronic low back pain, post–COVID-19 symptoms, anxiety/depression, or type 2 diabetes. The treatment focuses on how to initiate and maintain change, with a shift away from monitoring symptoms and toward an active approach to daily health-promoting microchoices. This short intervention has the potential of fundamentally changing the way we deliver health care to these patient populations, and hence, it could be a useful addition to the treatment armamentarium of the health care system, in the face of upcoming sociodemographic challenges.

## Abbreviations

BIPQ: Brief Illness Perception Questionnaire
CSQ: Client Satisfaction Questionnaire
MAR: missing at random
MI: multiple imputation
PUSH project: Project Development of Smart Health Solutions
WSAS: Work and Social Adjustment Scale
